# DNA extraction replicates improve diversity and compositional dissimilarity in metabarcoding of eukaryotes in marine sediments

**DOI:** 10.1371/journal.pone.0179443

**Published:** 2017-06-16

**Authors:** Anders Lanzén, Katrine Lekang, Inge Jonassen, Eric M. Thompson, Christofer Troedsson

**Affiliations:** 1 NEIKER-Tecnalia, Department of Conservation of Natural Resources, Bizkaia Technology Park, Derio, Spain; 2 Department of Biology, University of Bergen, Bergen, Norway; 3 Computational Biology Unit, Department of Informatics, University of Bergen, Bergen, Norway; 4 Sars International Centre for Marine Molecular Biology, University of Bergen, Bergen, Norway; 5 Uni Research Environment, Uni Research AS, Bergen, Norway; University of Guelph, CANADA

## Abstract

Human impact on marine benthic communities has traditionally been assessed using visible morphological traits and has focused on the macrobenthos, whereas the ecologically important organisms of the meio- and microbenthos have received less attention. DNA metabarcoding offers an alternative to this approach and enables a larger fraction of the biodiversity in marine sediments to be monitored in a cost-efficient manner. Although this methodology remains poorly standardised and challenged by biases inherent to rRNA copy number variation, DNA extraction, PCR, and limitations related to taxonomic identification, it has been shown to be semi-quantitative and useful for comparing taxon abundances between samples. Here, we evaluate the effect of replicating genomic DNA extraction in order to counteract small scale spatial heterogeneity and improve diversity and community structure estimates in metabarcoding-based monitoring. For this purpose, we used ten technical replicates from three different marine sediment samples. The effect of sequence depth was also assessed, and *in silico* pooling of DNA extraction replicates carried out in order to maintain the number of reads constant. Our analyses demonstrated that both sequencing depth and DNA extraction replicates could improve diversity estimates as well as the ability to separate samples with different characteristics. We could not identify a “sufficient” replicate number or sequence depth, where further improvements had a less significant effect. Based on these results, we consider replication an attractive alternative to directly increasing the amount of sample used for DNA extraction and strongly recommend it for future metabarcoding studies and routine assessments of sediment biodiversity.

## Introduction

Marine sediments harbour some of the most diverse biological communities and are vital to the maintenance of biogeochemical cycles and other ecosystem services. These ecosystems are relatively sensitive to anthropogenic disturbances, but there remains significant interest in exploitation of natural resources in such vulnerable environments [1–4]. Studies investigating the effects on benthic communities have traditionally focused on the macrobenthos, whereas meio- and microbenthos have received less attention, in spite of their importance to the marine ecosystem [5]. This is not surprising, given the diversity of microbial communities, coupled with the costs and logistical challenges of sampling and the time-consuming task of taxonomic identification [6,7]. Further challenges are the lack of easily identifiable morphological traits for many microbial taxa (i.e. cryptic diversity), a large number of undescribed organisms and a shortage of taxonomic expertise [8,9]. Thus, only a limited fraction of the diversity can typically be identified in traditional taxonomic studies, namely the macrobenthos, and to some extent the meiofauna. To overcome such challenges and limitations, the possibility of using environmental genomics to monitor a larger fraction of marine sediments is now a promising option [10–13].

DNA metabarcoding, the amplification and sequencing of molecular taxonomic markers from mixed samples (“barcodes”), offers a faster and more affordable way to study benthic diversity compared to morphological identification [14]. It can be applied directly to environmental DNA, to estimate presence or relative abundance of many established “indicator taxa” and to reveal previously hidden biodiversity of the meio- and microbenthos. However, taxonomic identification of the resulting molecular operational taxonomic units (OTUs) may be crude, partly due to technical limitations, but mainly because they do not constitute established or previously encountered species [15–17]. Nonetheless, such data can be critical for understanding anthropogenic impacts, and relating them to ecosystem dynamics, in a manner not feasible using microscopy. This has already been demonstrated in assessing microbial eukaryote communities, using the small subunit ribosomal RNA gene (SSU rRNA) as a marker (reviewed in [11], but also see [9,13,18,19]). As yet, we have only skimmed the surface of microbial benthic diversity [20], many basic aspects of experimental design remain poorly known, and the methodologic pipelines are not sufficiently standardised.

The relationship between the number of individuals (or biomass) of a particular species with the abundance of its rRNA gene, in sampled sediment material, differs widely between taxa. The rRNA gene copy number [21,22] and PCR biases [23,24] are variables that influence this relationship. The former tends to increase with cell size [25,26] and genome size [27], although some organisms such as ciliates contain exceptional numbers of rRNA operons [22,26]. Obviously, the total number of rRNA gene copies present in a multicellular organism individual also depends on its cell number. The number of rRNA gene copies of a taxon in environmental DNA can thus be considered a reasonable proxy for biomass. However, sample material used for DNA extraction typically contains whole living microorganisms, whereas macrobenthos are traceable mainly through dead tissue and extracellular remains.

In spite of inherent biases, metabarcoding can generally be considered semi-quantitative, and useful for comparing abundances between samples [28–30]. Thus, given representative samples of environmental DNA, we can assume that the relative change in sequence read numbers between species will reflect the relative change in abundance between species, but not the actual number of individuals in any given sample. This provides a method to describe population dynamics in the sediments. However, this core assumption is challenged by spatial heterogeneity or “patchiness”, particularly when sample sizes are small relative to organism size [31,32]. It is also challenged by random errors inherent in DNA extraction, particularly in the presence of inhibiting compounds [33]. This can be improved by increasing spatial replication [34] or sample size, improved mixing of sample material, using more material for DNA extraction [35], or by using several technical replicates of DNA extraction [36,37]. The latter may be the most straightforward way to improve accuracy, considering the small spatial scales at which much of heterogeneity occurs. While sufficient spatial replication remains fundamental, this can also help to control replication of other more reproducible measurements.

As opposed to running replicate PCRs for library preparation, DNA extraction is typically not replicated in metabarcoding studies, although many previous studies suggest the need for such replication. Brannock and Halanych [32] found differences in meiofaunal community structure to be relatively high between extraction replicates from sediments and recommended that such replication be carried out for metabarcoding and that replicates are not pooled prior to sequencing in order to assess heterogeneity. Based on DNA fingerprinting of soil microbes, Feinstein et al. [37] recommended to use at least three extraction replicates per sample. Penton et al. [35] later demonstrated that larger extraction volumes can compensate for this heterogeneity to some extent, although this was not explicitly compared to replication.

Here, we evaluate the effect of using technical replicates of DNA extraction when assessing biodiversity by metabarcoding of eukaryotes in marine sediments. To this end, we used ten replicates each from three well-mixed bulk sediment samples with different characteristics. We compare the effect of technical replication to increased sequence depth, both for estimating alpha diversity and the ability to correctly separate samples based on community dissimilarity. Samples were collected in connection with a study evaluating the use of metabarcoding for routine monitoring of sediment biodiversity adjacent to offshore drilling platforms, on the Norwegian continental shelf in the North Sea [13].

## Materials and methods

### Study site, samples, sample processing and DNA extraction

Sediments with contrasting characteristics from three different offshore oil fields in the Troll-Oseberg region (Region III) on the Norwegian continental shelf were sampled as part of ongoing environmental monitoring program in 2010 by DNV for Statoil Petroleum AS [38] in conjunction with a more extensive metabarcoding study [13]: (1) a sediment composed of fine sand from Oseberg C station 7 (“Fine Sand”), (2) a sediment composed of coarse sand from Huldra station 9 (“Coarse Sand”), and a clay-dominated sediment from Fram station A2-01 (“Clay”). Samples were collected in compliance with the Norwegian activity regulations ("Aktivitetsforskriften §52"), the Norwegian framework regulations ("Rammeforskriften § 48") and "Guidelines for offshore environmental monitoring: The petroleum sector on the Norwegian Continental Shelf" (M-300). Such monitoring is legally required by all companies undertaking extraction activities on the Norwegian continental shelf. During sampling, aliquots for molecular analysis were donated to the authors for research purpose by Statoil as part of the NFR funded project 190265/S4. Sample collection, processing, genomic DNA extraction and physicochemical analyses were carried out as described previously [13], except the ten extraction replicates were not pooled prior to PCR amplification. Briefly, 50–100 g of sediment was fixed with 96% ethanol, stored at -20°C and centrifuged at 6000x g in order to remove ethanol. Samples were then mixed thoroughly using a sterile metal spatula prior to collecting ten replicates of 0.5 g sediment material for genomic DNA extraction using PowerSoil® kits (MO BIO Laboratories Inc., Carlsbad CA), as recommended for marine sediments [33].

### Amplicon library preparation and sequencing

**Fig 1:**
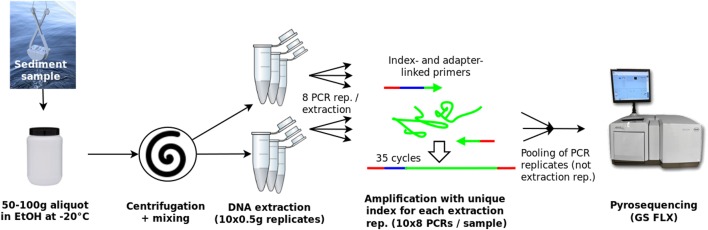
Sample processing including technical replication of DNA extraction and amplicon library preparation.

Amplicon library preparation was carried out as described previously [13], with the exception that PCR amplification was carried out individually for each of the ten extraction replicates per sample (Fig 1). For each of the ten extraction replicates, eight replicate PCRs were carried out and later pooled, resulting in a total of 30 sample-replicates, each indexed with a unique sequence tag (i.e. one for each independent extraction, for each of the three samples). Briefly, the V4-V5 region of the eukaryotic SSU rRNA gene (18S) was amplified using index- and adapter-linked primers F566 and R1200 [39], 2.5 μl extracted DNA, 1 μg/μl Bovine Serum Albumin, and HotStar Taq Master Mix (Qiagen) to a volume of 25 μl, for 35 cycles. Concentrated amplicons were purified using Agencourt AMPure XP (Beckman Coulter Inc.) and DNA concentrations thereafter determined using Quant-iT^TM^ PicoGreen® dsDNA quantification kit (Invitrogen) and an ND3000 fluorospectrophotometer (Nanodrop Technologies Inc.). Finally, amplicon libraries were pooled in equimolar amounts and sequenced using a Genome Sequencer FLX (454 Life Sciences) with Titanium reagents, at the Norwegian Sequencing Center (University of Oslo, Norway). Sequence data is available from the NCBI Read Archive (Bioproject accession PRJNA225939, Sample accession SRS507106).

### Sequence data processing

Demultiplexed flowgram data (SFF files) was processed using AmpliconNoise and Perseus [40] in order to correct artifacts from sequencing and PCR (including chimeric sequences), as described in Lanzén et al. [13]. Resulting “denoised” sequence datasets were subjected to maximum distance (complete linkage) hierarchical clustering using NDist and Fcluster [40], retaining read abundance information, resulting in an Operational Taxonomic Unit (OTU) frequency table with 2% maximum divergence (98% minimum similarity, see [13]; available as S1 Data). Relative OTU abundances were calculated by dividing dataset specific OTU read abundances with the total number of denoised reads for the dataset. Singleton OTUs were retained in order to maintain each dataset as a representative sample of the underlying OTU abundance distribution.

Representative OTU sequences were taxonomically classified using CREST (SilvaMod) with default parameters except for a minimum bitscore of 100 [41]. Based on classification, the OTU distribution table was annotated in order to allow filtering targeting only the metazoan (macro- and meio- zoobenthos), or protist OTUs (all other OTUs classified as eukaryotes, except fungi or Viridiplantae; representing the meio- and microbenthos; see S2 Data).

In order to compare individual genomic DNA extraction replicates with the use of multiple pooled replicates, pooling was carried out *in silico* using R, resulting in an alternative OTU table combining all reads from replicates of each of the three samples.

### Statistical analyses estimating influence of replication and sequence depth

Rarefaction analysis, sub-sampling and calculation of Shannon diversity were calculated using R with packages *vegan* and *phyloseq* [42,43]. Datasets with a sequence depth lower than 15,000 reads were excluded from analysis where the effect of sequence depth was evaluated using rarefaction or repeated sub-sampling. Silhouette scores [44] were calculated using the R package *cluster* [45].

For rarefaction analysis of richness including all OTUs, average and standard error among the rarefied richness of all individual replicates were calculated (rarefied richness being the expected species richness in random sub-samples of a given number of reads; as described in [46] and implemented in vegan's *rarefy* function). This was carried out at sequence depth intervals of 500 or 1000 reads, excluding replicates with fewer reads. For the *in silico* pooled replicates, standard errors were instead estimated directly using the function *rarefy* as described in [47].

For Shannon diversity estimates, or taxonomic subsets (metazoa or protists), repeated (100x) random sub-sampling of OTU distribution tables was carried out for each replicate at sequence depth intervals of 500 reads, using the function *rrarefy*. Taxon-specific results for metazoa or protists were derived by first sub-sampling all OTUs to the specified number of reads, thereafter filtering retained OTUs based on classification, so as to estimate the effect of total read depth from data derived with eukaryotic universal primers, while targeting only a taxonomic subset. Relative richness gain from pooling replicates was calculated for each interval as the pooled rarefied richness or Shannon diversity divided by the corresponding average for single-replicate estimate.

In order to estimate the influence of the number of genomic DNA extraction replicates on alpha-diversity, independently from sequence depth, the following repeated sampling scheme was carried out: *r* reads were randomly sub-sampled and merged from each of *n* randomly selected extraction replicates, with *n* ranging from one to ten (the total number of replicates available for each sample) and *r = 15*,*000/n* so that total merged sequence depth was kept constant at 15,000 reads. This procedure was repeated 100 times for each value of *n* and for each sample. Taxonomic filtering was carried out after this sub-sampling, in order to compare the influence of all OTUs, to protist or metazoan OTUs (see Sequence data processing above).

The effect of read number (sequence depth) on accuracy of community dissimilarity measures based on single genomic DNA extraction replicates, was simulated by varying sequence depth from 500 to 15,000 (at 500 read intervals). For each depth, replicates were sub-sampled, excluding those with lower sequence depth. Two types of analyses were then performed, both repeated 100x per simulated depth. First, the mean within-sample dissimilarity was calculated for each sample (Bray-Curtis and Jaccard, based on relative abundances and presence/absence of OTUs, respectively). Second, silhouette scores [44] were calculated, based on Bray-Curtis dissimilarities, estimating the consistency of the three “correct” clusters, i.e. extraction replicates originating from each sample forming separate clusters.

To simulate the effect of replication on community dissimilarity, independent from sequence depth, a procedure similar to the corresponding estimates for alpha-diversity was carried out. However, *n* replicates from each sample were randomly selected without re-sampling, sub-sampled to *r* reads, and merged to a “pseudo-replicate”. This procedure was then repeated until no more replicates were available, i.e. 10/*n* times (rounded down to the nearest integer). In order to simulate a constant total sequence depth of 15,000, *r* was therefore chosen as 15,000*/n*. Resulting pseudo-replicates (minimum 2), were then used to calculate mean silhouette scores as described above. This simulation was repeated 100x for each *n* (ranging from 1 to 5). For all simulations of sample dissimilarity, taxonomic filtering was also performed, resulting in separate estimates for protists and metazoa. R code is available on request.

## Results

### Sample overview

Pyrosequencing resulted in over half a million reads. Less than 0.4% of reads, or 23% of OTUs, were singletons (see S1 Table for details). These were retained in subsequent analyses. Reads were evenly shared between the three samples (“Fine Sand”, “Coarse Sand” and “Clay”), each representing a dataset of *in silico* pooled genomic DNA extraction replicates (hereafter named “pooled samples”). However, intra-sample variation in sequence depth was high, varying between 2674 to 36982 reads per replicate (S1 Table). This likely explains the high intra-sample variation in OTU richness as well, because it was strongly correlated with the number of reads (sequence depth) (p = 2x10^-3^, r = 0.54). This, in turn indicates that sequence depth in individual replicates was not sufficient for a complete diversity census. To compensate, we only considered expected diversity at a given sequence depth, using rarefaction or repeated random sub-samples. Nonetheless, pooled samples appeared relatively unique, with only 1409 OTUs of 6823 (21%) shared between two or more samples. Further, Clay was 40% less OTU rich than the other two samples, although its Shannon diversity was similar (S1 Table).

Protists accounted for a majority of reads from fine- (74–88%) and coarse (66–86%) sand, and about half (21–58%) from the Clay sample, representing 60–68% of all OTUs. Metazoa showed greater heterogeneity, with relative abundance ranging from 8–20%, 3–30%, and 32–74% in Fine Sand, Coarse Sand and Clay respectively, representing 7–9% of all OTUs per sample (S1 Table).

### Effect of sequence depth and genomic DNA extraction replications on diversity estimates

**Fig 2:**
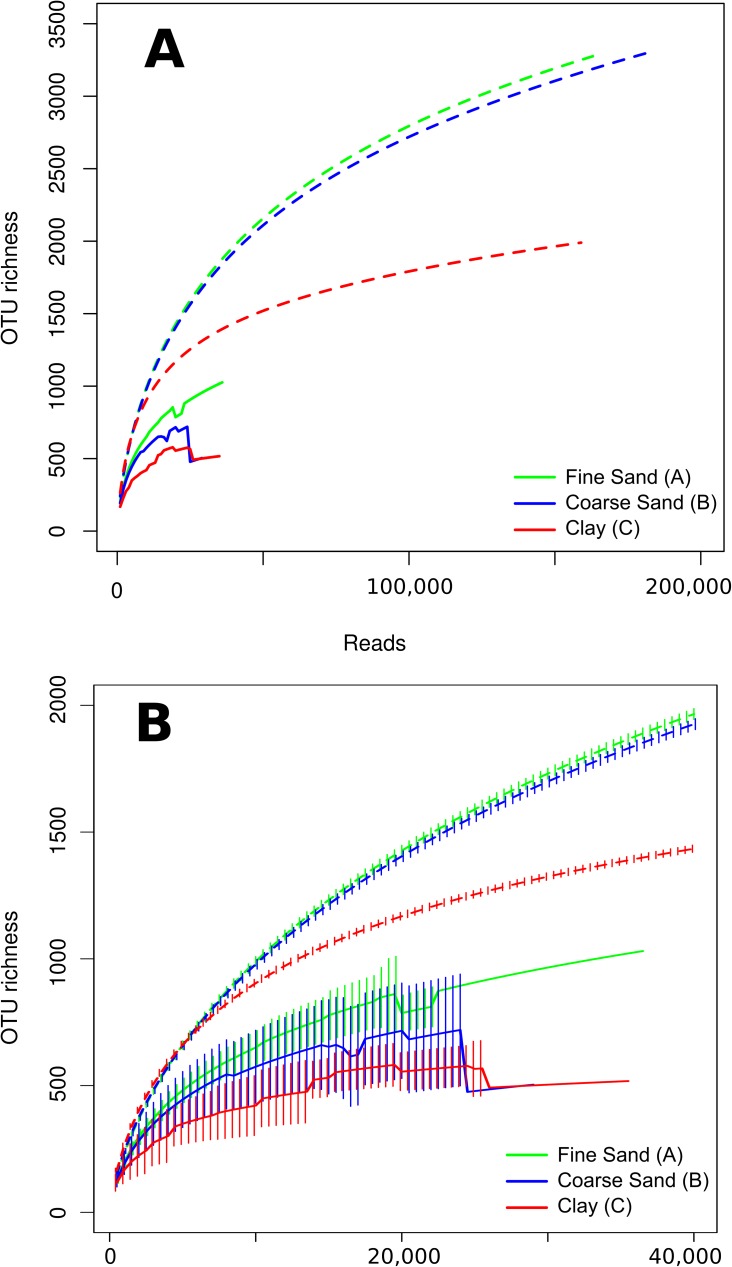
Expected OTU richness as a function of sequence depth (rarefaction). *In silico* pooled replicates are shown as dashed lines and individual extraction replicates as solid lines. In panel A richness up to the maximum read number for pooled replicates and in B the same rarefaction curves up to maximum read number for individual replicates. Error bars (in B) represent standard error, for pooled samples calculated as described in Heck et al [47].

**Fig 3:**
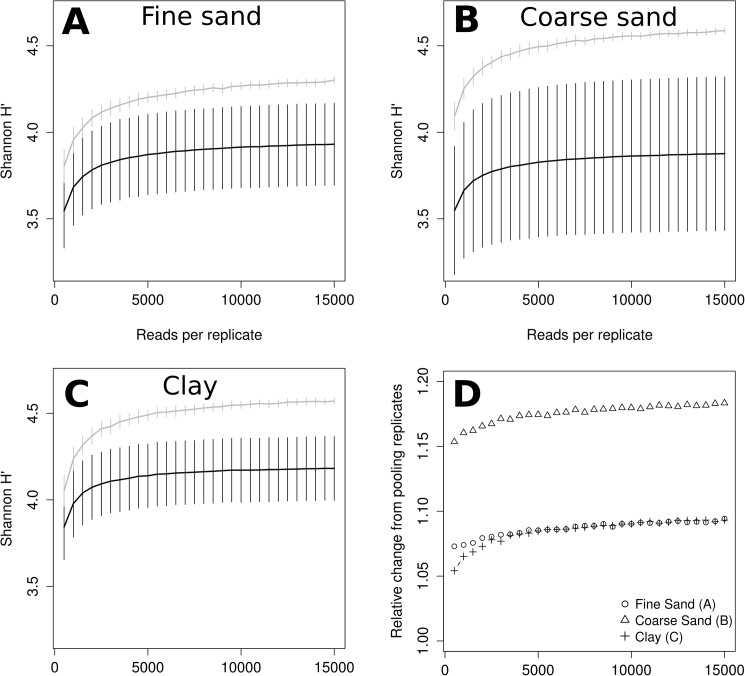
Rarefaction comparing expected OTU richness of pooled samples (grey lines) and individual replicates (black lines). Only replicates with more than 15,000 reads were included from the samples Fine Sand (A; n = 5), Coarse Sand (B; n = 7) and Clay (C, n = 5). Panel D shows expected richness in pooled samples compared to mean expected replicate richness. Error bars represent standard error, for pooled samples calculated as described in Heck et al [47].

A rarefaction analysis including all pooled samples and individual replicates confirmed that sequencing in individual replicates was far from exhaustion, indicating that over 150,000 reads per sample would be needed to approximate the full diversity of any sample, since the slopes of rarefaction curves at this read depth ranged between 0.003–0.007 (Fig 2). In addition, standard error among replicates was too high to support significant differences between the three samples, whereas for pooled datasets Clay showed significantly lower richness above approximately 15,000 reads (Fig 2). Importantly, this analysis indicates that pooled samples, combining the ten extraction replicates, showed significantly higher richness even at sequencing depths reached by individual replicates. This is illustrated in Fig 3, where the corresponding rarefaction was carried out to a maximum of 15,000 reads (excluding all replicates with fewer reads) to estimate expected richness more consistently. Thus, we included five replicates each from Fine Sand and Clay, and seven from Coarse Sand. This illustrates that replicating extraction can prevent underestimation of alpha-diversity, and, if spatial replicates are used, overestimation of beta-diversity.

Replicating genomic DNA extraction increased richness most for the Clay sample, with almost twice as many OTUs recovered compared to the average individual replicate at 15,000 reads (Fig 3). Clay and Fine Sand showed similar patterns for relative gain in richness (1.8 and 1.6 respectively), correlating to sequence depth with decreasing but positive slope at 15,000 reads (Fig 3). Similar trends were observed when limiting OTUs to protists (S1 Fig), whereas for metazoa the difference between pooled samples and individual replicates was higher for Clay and Coarse Sand and lower for Fine Sand (S2 Fig).

Singletons were retained in order to not skew the underlying OTU abundance distribution and had relatively minor effect at this sample depth, increasing rarefied OTU richness at 15,000 reads by <6%, or 71 OTUs (S3 Fig).

**Fig 4:**
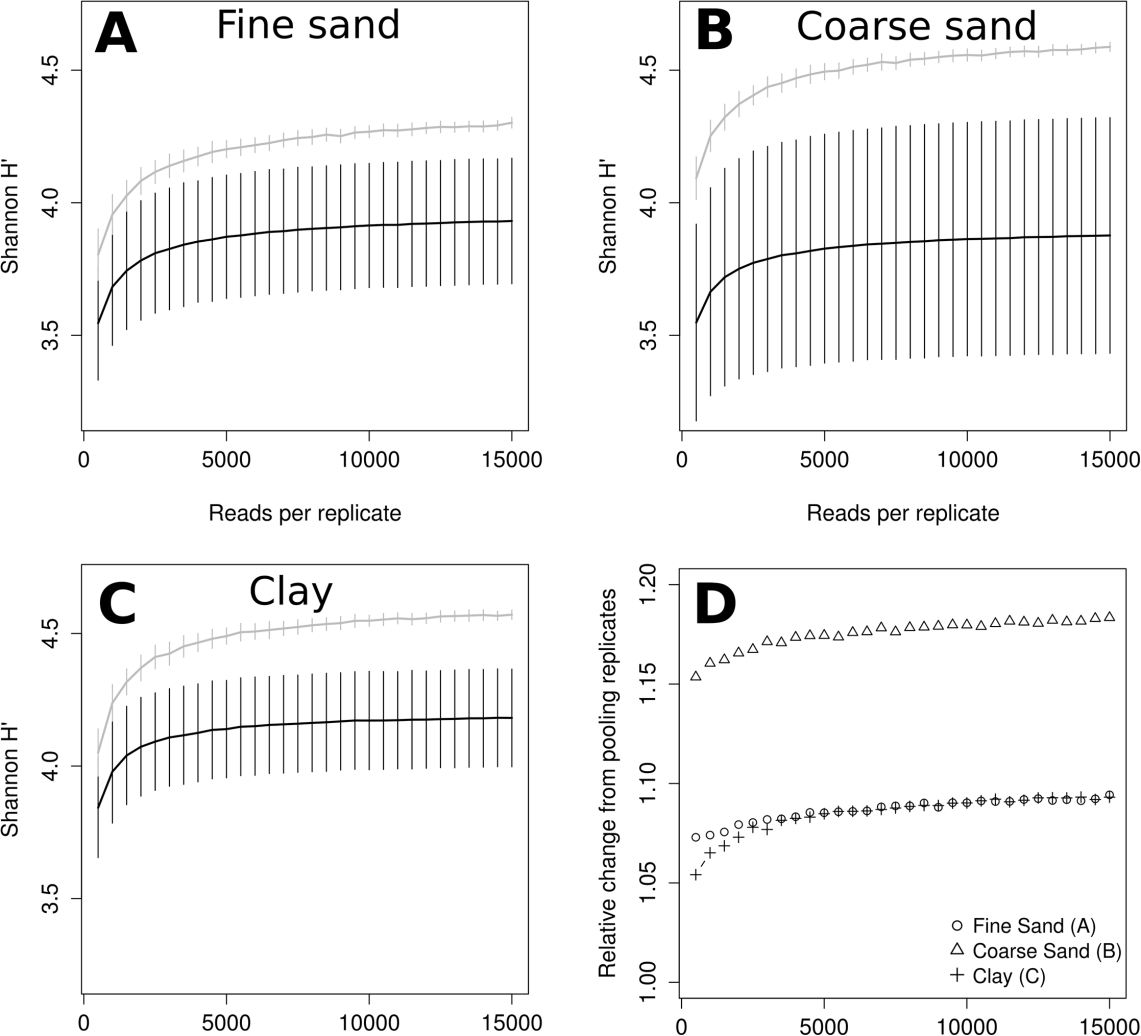
Rarefaction comparing Shannon diversity of pooled samples (grey lines) and individual replicates (black lines). Expected Shannon diversity as a function of read sequence depth was estimated using repeated random sub-sampling from replicates with >15,000 reads from Fine Sand (A; n = 5), Coarse Sand (B; n = 7) and Clay (C, n = 5). Panel D shows expected richness in pooled samples compared to mean expected replicate richness.

Estimated Shannon diversity also appeared to increase clearly with sequence depth up to about 5,000 reads (Fig 4). However, relative standard error between replicates appeared higher compared to that of rarefied OTU richness, and the gain in estimated diversity was lower (Fig 4; note however, that Shannon diversity scales with the logarithm of richness). The effect was stronger in Coarse Sand, likely due to higher variation between replicates (Fig 4).

**Fig 5:**
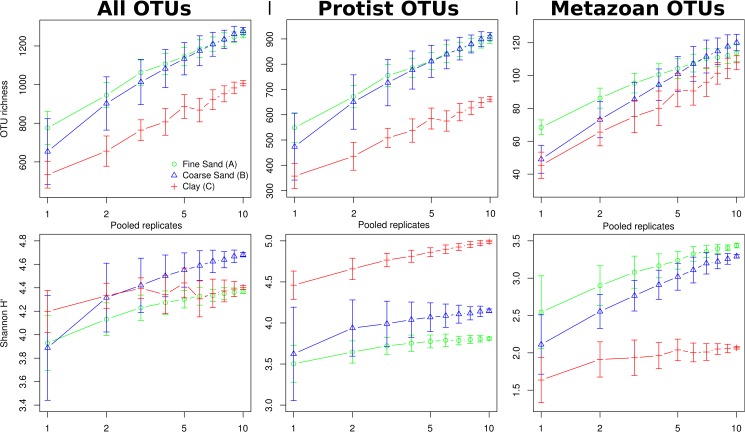
Effect of pooled extraction replicate number on OTU richness and Shannon diversity. Mean diversity estimates based on repeated (i = 100) random selection of replicates to pool followed by random sub-sampling of OTUs to a total sequence depth of 15,000 reads per sample (divided equally between selected replicates). Filtering to protist or metazoan OTUs was carried out after sub-sampling. Error bars represent the standard error of values from the bootstrapping procedure described above.

In order to estimate the effect of the number of DNA extraction replicates independent of sequencing depth, we also varied the number of replicates from which OTU distribution tables were randomly and repeatedly (100x) sub-sampled, while keeping sequencing depth constant at 15,000 reads, evenly distributed between the randomly selected replicates. According to this simulation, both richness and Shannon diversity increased in a roughly log-linear fashion with the number of replicates (Fig 5). However, some degree of saturation (decreased slope) can be seen before reaching 10 pooled replicates in several samples for protist OTU richness or Shannon diversity.

### Effect of sequence depth and DNA extraction replications on sample divergence

**Fig 6:**
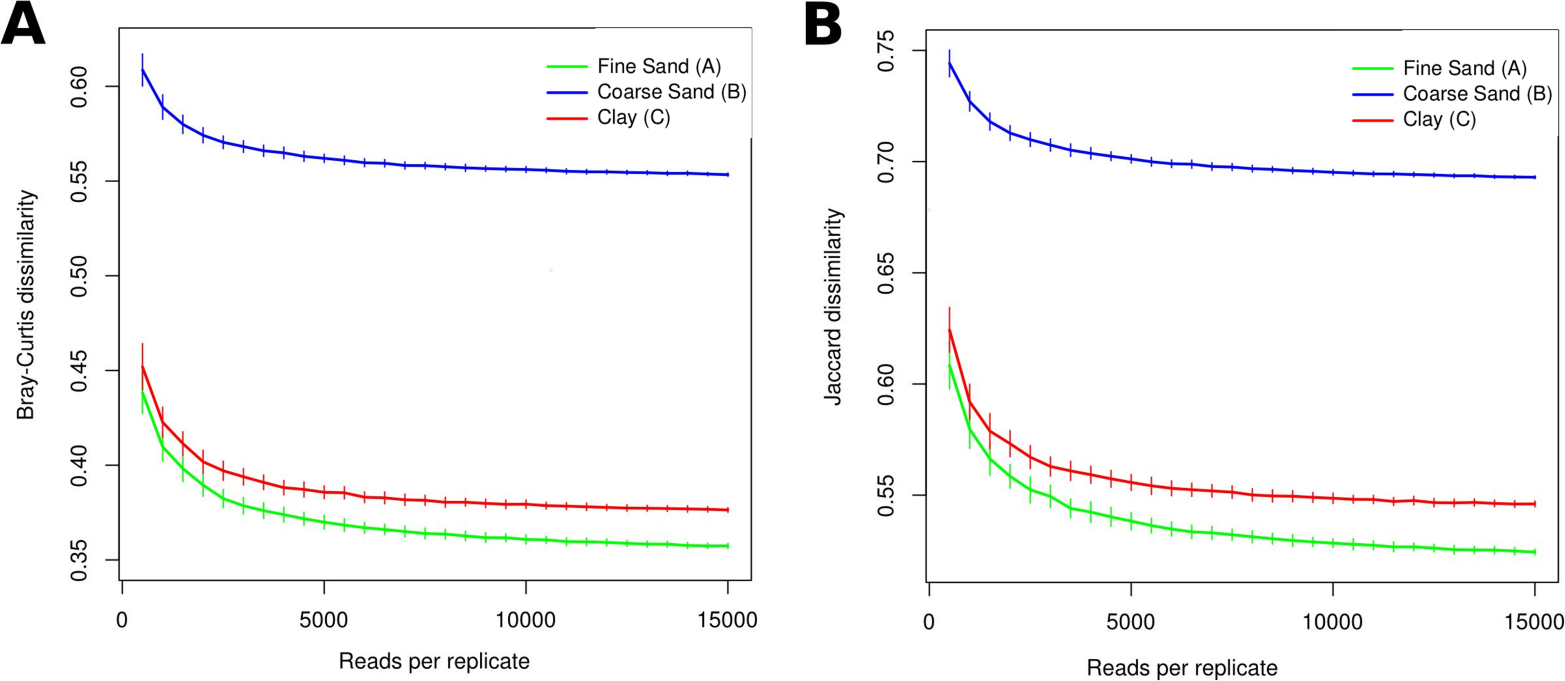
Effect of sequence depth on intra-sample divergence. Divergence is expressed as (A) Bray-Curtis and (B) Jaccard dissimilarity between repeated (i = 100) random sub-samples of all replicates with >15,000 reads. Error bars represent bootstrapped standard errors.

In addition to alpha-diversity estimates (OTU richness and Shannon diversity), we evaluated the effect of sequencing depth on intra-sample divergence, measured as dissimilarity between all replicates belonging to the same sample (replicates <15,000 reads excluded). Resulting dissimilarities decreased sharply with sequence depth until about 5000 reads, after which the effect was more subtle, both for relative-abundance based Bray-Curtis and presence/absence based Jaccard dissimilarity (Fig 6). Divergence between replicates was significantly higher in Coarse Sand, consistent with a higher variation in alpha-diversity estimates.

**Fig 7:**
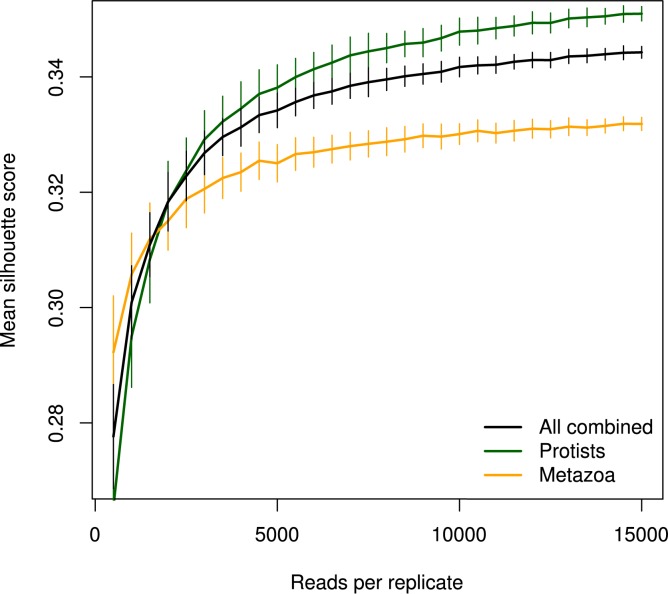
Effect of sequence depth per replicate on mean silhouette score, representing the separability of replicates based on sample of origin, based on repeated sub-sampling of OTUs. Filtering to protist or metazoan OTUs was carried out after sub-sampling. Error bars represent bootstrapped standard errors.

Silhouette scores [44] were applied for measuring the separability of replicates originating from different samples. These scores (ranging between zero to one) measure both cohesion and separation, i.e. how similar each replicate is to other replicates of the same origin compared to replicates of non-related origin. Using the same sub-sampling procedure and sequence depth intervals as described above, mean silhouette scores and their standard error were calculated. Following a similar but inversed trend as intra-sample divergence, silhouette scores improved sharply from 500 to 5000 reads, and thereafter more subtly with increasing sequence depth (Fig 7). Limiting analysis to protist OTUs appeared to improve sample separation compared to also including metazoa (Fig 7).

**Fig 8:**
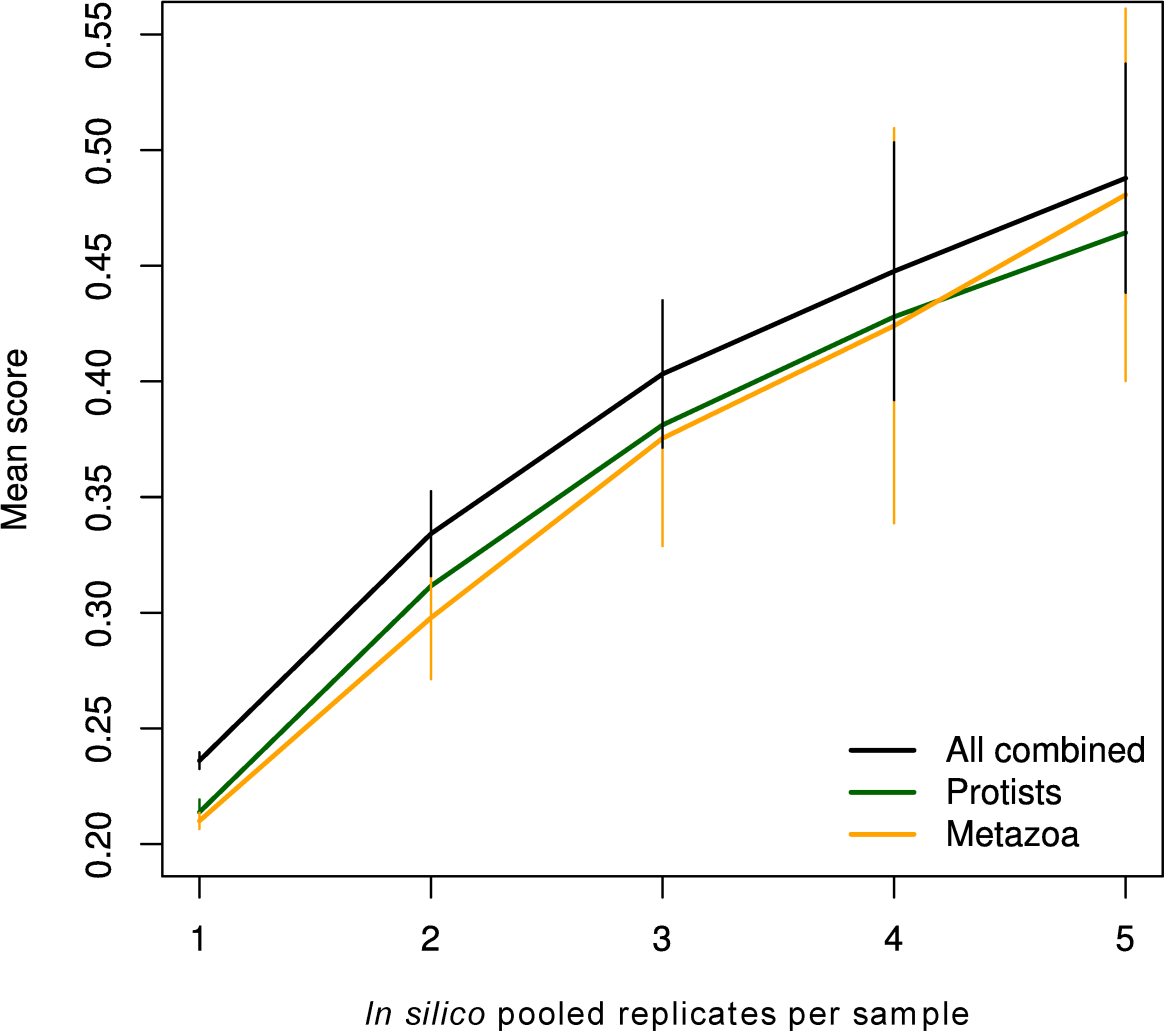
Effect of pooled extraction replicate number on silhouette score. Mean silhouette scores were calculated based on repeated random selection of replicates to pool and sub-sampling of OTUs to a total sequence depth of 15,000 reads per sample (divided equally between selected replicates). Filtering to protist or metazoan OTUs was carried out after sub-sampling. Error bars represent bootstrapped standard errors.

We also utilised silhouettes to simulate the effect of degree of replication of DNA extraction on separability of samples. This demonstrated a clear increase in mean score as single replicates were used, then two replicates randomly pooled, and finally five pooled into two “replicate pools” per sample (Fig 8). Reads per sample were maintained at 15,000 to counteract influence of sequence depth. Thus, each replicate contributed 7,500 reads or less, below the mid-point in the previous analysis (Fig 7). A considerably higher silhouette score was reached already when pooling 3 extraction replicates, indicating the strong potential of incorporating such replication in experimental design to improve accuracy.

## Discussion

It is well established that benthic communities display considerable small-scale spatial heterogeneity, ranging from scales of centimetres to several meters [48–50]. Several metabarcoding studies have shown that this can be alleviated to some extent by carrying out several replicate DNA extractions [31,32,51]. The results presented here based on three distinctly different sediment samples (Fine Sand, Coarse Sand and Clay) strongly support this practice and demonstrate that replication of genomic DNA extraction can improve OTU-based estimates of alpha-diversity as well as dissimilarity. Further, we show that this effect is independent of read number and thus cannot be achieved by increasing sequencing depth alone. This also suggests that it was not a consequence of sequencing and PCR errors, expected to artificially inflate diversity estimates of pooled as well as individual replicates to the same extent at a given number of reads. However, it is possible that intra-sample divergence increased as a consequence of differences between the indices linked to the primers used for amplicon library preparation. It has been demonstrated that this may induce PCR bias leading to Bray-Curtis dissimilarities as high as 0.69 between technical replicates [52].

The positive effect of replication for recovering alpha-diversity increased with sequence depth and was more pronounced for metazoa compared to protists (Figs 2 and 3, S1 Fig and S2 Fig). This is consistent with metazoa having a more heterogeneous distribution due to their larger size. In order to homogenise samples, mixing was carried out prior to sub-sampling for DNA extraction using a metal spatula. Taken together, our results suggest that this mixing was not sufficient to compensate for small-scale heterogeneity, but can be alleviated by replicating DNA extraction. Indeed, mixing larger samples before subsampling for DNA is possible and will likely reduce heterogeneity. However, in large-scale environmental assessments with high sample numbers, this procedure is prohibitive due to the tedious nature of the process. The extent to which random variation inherent to the DNA extraction procedure itself contributed to differences between replicates was not possible to quantify.

Using three DNA extraction replicates and SSU rRNA primers specific to Foraminifera, Lejzerowicz et al. [31] found that each replicate contained on average 58% of the total sample diversity. In spite of using approximately three times higher sequencing depth per replicate (54,000 reads), this is lower than the expected difference in protist OTU richness between pooling three replicates of 15,000 reads each in any of the three samples investigated here (Fig 5). This may be a consequence of Foraminifera having a more heterogeneous spatial distribution than the average protist, consistent with their larger size. Further, it was noted that OTUs with a higher relative abundance tended to be shared by all three replicates more often than rare OTUs [31]. This is consistent with our prediction of the relative gain in OTU richness (Fig 3) increasing with sequence depth, as a higher read number will cover a larger proportion of rare OTUs.

By comparing triplicate cores from the same box-core or multicore, Guardiola et al. [19] measured intra-sample Bray-Curtis and Jaccard dissimilarity values (0.6 and 0.7 respectively) comparable to the most heterogeneous sample analysed here (Coarse Sand) at corresponding sequencing depth (11,000 reads). However, these results are not directly comparable since a different (v7) and shorter (100–110 bp) region of the SSU rRNA gene was used [19]. Further, most samples were less diverse than any single replicate studied here and the majority of reads as well as OTUs were metazoan, as opposed to distributions in our samples. Even in the replicates where metazoa dominated the reads, they only contributed 18% of OTU richness (S1 Table).

Using simulations and two metabarcoding datasets, including a survey of nine earthworms species, Ficetola et al. [51] explored the use of occupancy models for predicting detection probability and optimisation of replication levels. Although extraction replicates from their earthworm survey did not appear significantly different, detection probabilities of individual species ranged from 0.52 to 0.96, indicating that replication could improve accuracy. However, it was noted that occupancy models do not work when targeted diversity is too high or cryptic, both of which apply here [51].

Apart from replication, another approach that may reduce heterogeneity is increasing the amount of sample from which DNA extraction is carried out, as has been assessed by Brannock and Halanych for marine meiobenthos [32]. The MoBio PowerSoil® (same as in this study) was used for 0.3 g samples, whereas MoBio PowerMax® kits were used for 5 and 10 g samples. These kits use identical chemistry and protocols except for scale. For the largest 200 and 400 g samples, PowerSoil® kits were used, but elutriation was carried out prior to extraction, with a 45 μm sieve. Two extraction replicates were consistently used. Different extraction amounts did not result in significantly different diversity estimates [32]. This indicates that replicated smaller extractions, as suggested here, is more cost efficient for improving such estimates. However, it appears that the 5 g extractions resulted in more consistent results across replicates. They also noted a more consistent taxonomic composition in elutriated vs. non-elutriated samples [32].

A similar study comparing extraction methods for metabarcoding of bacteria and fungi concluded that methods using more soil for extraction had significantly higher diversity estimates and lower intra-sample divergence [35]. These results may differ from ours because of different characteristics of soil versus marine sediments, and the focus on prokaryotes and fungi, possibly leading to increased micro-habitat diversity.

A recent evaluation of metabarcoding of soil fungi found that that alpha-diversity estimates did not increase significantly with the number of PCR replicates used, as opposed to increasing sequence depth [53] or, as assessed here, using DNA extraction replicates (Fig 5). This may be explained by the fact that PCR replication cannot counteract spatial heterogeneity, whereas DNA extraction can. Further, it was noted that alpha-diversity and sample dissimilarity was highly consistent between the two sequencing platforms 454 Pyrosequencing and Illumina MiSeq [53]. Given that MiSeq offers higher sequence depth at a significantly lower cost compared to the now retired pyrosequencing platform, it would be very interesting to utilise it to repeat the experiment described here.

In conclusion, our analyses demonstrated that both sequencing depth and extraction replicates can improve the coverage of targeted organisms, estimates of alpha diversity, and the ability to separate samples with different characteristics. These effects did not appear to stabilise at any given number of replicates, i.e. including more than ten (or five) extraction replicates per sample would likely improve coverage, diversity estimates and separability further. Furthermore, no sequencing depth was found beyond which diversity estimates or separability ceased to increase. We can also expect sequencing and PCR errors to inflate alpha-diversity estimates dependent on sequence depth, and thus that the slope of rarefaction curves will never reach zero, even if all samples were sequenced exhaustively. Thus, it remains very challenging to answer the question of how many replicates or sequence reads are “enough”. The appropriate level of replication will depend on the aim of the study, the number of biological replicates and the degree of divergence between communities of interest. If possible, we recommend preliminary sequencing to be carried out from a selection of samples, to guide the experimental design of larger studies, where individual replicates are sequenced separately rather than pooled. Thus the degree of intra-sample divergence can be estimated, allowing for a cost-benefit analysis where the accuracy of community dissimilarity and diversity estimates are optimized against the cost per sample.

## Supporting information

S1 Data OTU distribution table. (TSV)

S2 Data Taxonomic classification and category (protists, metazoa or unknown) for representative OTU sequences. (TSV)

S1 Table Overview of sequence depth, richness and taxonomic composition (protists and metazoa) for all replicates and pooled samples. (XLSX)

S1 Fig Rarefaction analysis of protist OTU richness for pooled samples (grey lines) and individual replicats (black lines). Mean and standard error of expected richness was calculated based on repeated random sub-samples at each read interval from replicates of the samples Fine Sand (A; n = 5), Coarse Sand (B; n = 7) and Clay (C, n = 5), after taxonomic filtering to include only protist OTUs. Panel D shows expected richness in pooled samples compared to mean expected replicate richness.
(TIF)

S2 Fig Rarefaction analysis of metazoan OTU richness for pooled samples (grey lines) and individual replicates (black lines). Mean and standard error of expected richness was calculated based on repeated random sub-samples at each read interval from replicates of the samples Fine Sand (A; n = 5), Coarse Sand (B; n = 7) and Clay (C, n = 5), after taxonomic filtering to include only metazoan OTUs. Panel D shows expected richness in pooled samples compared to mean expected replicate richness.
(TIF)

S3 Fig Rarefaction analysis of pooled samples illustrating the effect of retaining singletons. Rarefaction curves using solid lines represent the studied dataset where singletons were retained (and correspond to Figs 1 and 2), whereas dashed lines result from rarefaction curves after removing singletons.
(TIF)
