# Correction: DNA extraction replicates improve diversity and compositional dissimilarity in metabarcoding of eukaryotes in marine sediments

**DOI:** 10.1371/journal.pone.0192337

**Published:** 2018-01-30

**Authors:** Anders Lanzén, Katrine Lekang, Inge Jonassen, Eric M. Thompson, Christofer Troedsson

Fig 3 is incorrect. The authors have provided a corrected version here.

**Fig 3:**
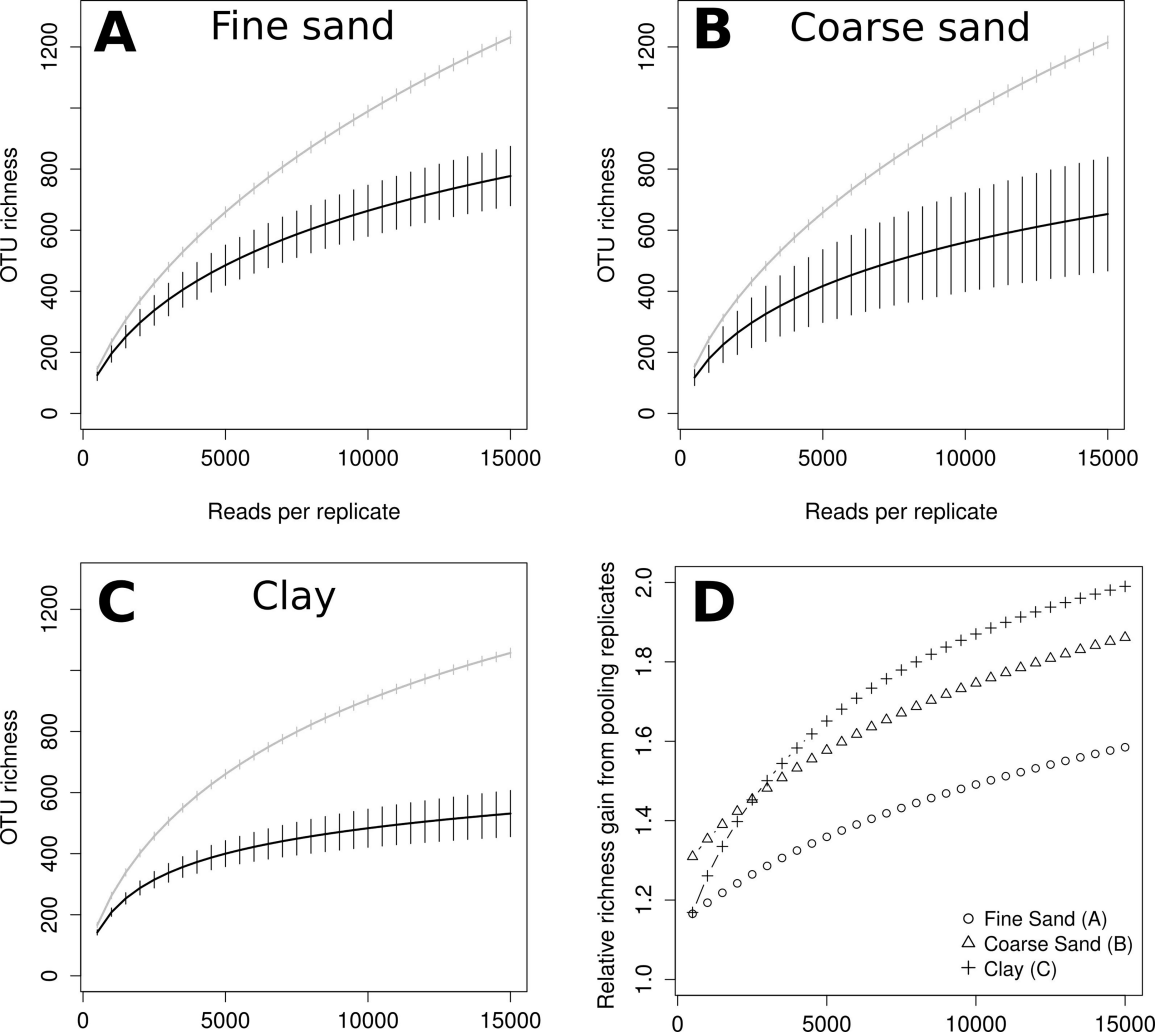
Rarefaction comparing expected OTU richness of pooled samples (grey lines) and individual replicates (black lines). Only replicates with more than 15,000 reads were included from the samples Fine Sand (A; n = 5), Coarse Sand (B; n = 7) and Clay (C, n = 5). Panel D shows expected richness in pooled samples compared to mean expected replicate richness. Error bars represent standard error, for pooled samples calculated as described in Heck et al [47].

47. Heck KL, van Belle G, Simberloff D. Explicit Calculation of the Rarefaction Diversity Measurement and the Determination of Sufficient Sample Size. Ecology. 1975;56: 1459–1461.
