# Supplementary figures and images for: DNA extraction replicates improve diversity and compositional dissimilarity in metabarcoding of eukaryotes in marine sediments
Source: PLoS One. 2017 Jun 16;12(6):e0179443. doi: 10.1371/journal.pone.0179443 (PMC5473592; doi:10.1371/journal.pone.0179443)

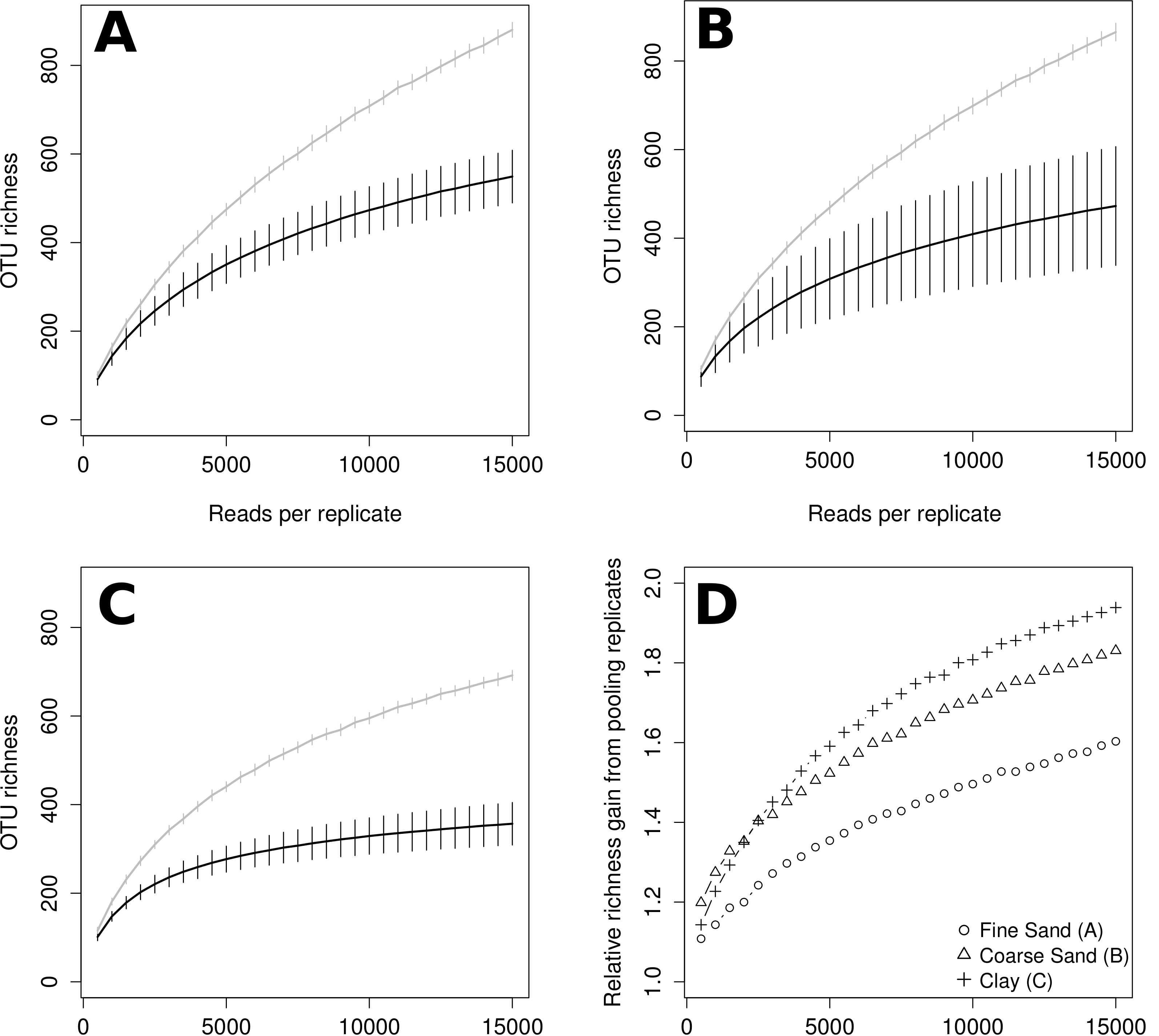

Supplement: S1 Fig — Mean and standard error of expected richness was calculated based on repeated random sub-samples at each read interval from replicates of the samples Fine Sand (A; n = 5), Coarse Sand (B; n = 7) and Clay (C, n = 5), after taxonomic filtering to include only protist OTUs. Panel D shows expected richness in pooled samples compared to mean expected replicate richness. (TIF) [file pone.0179443.s004.tif]

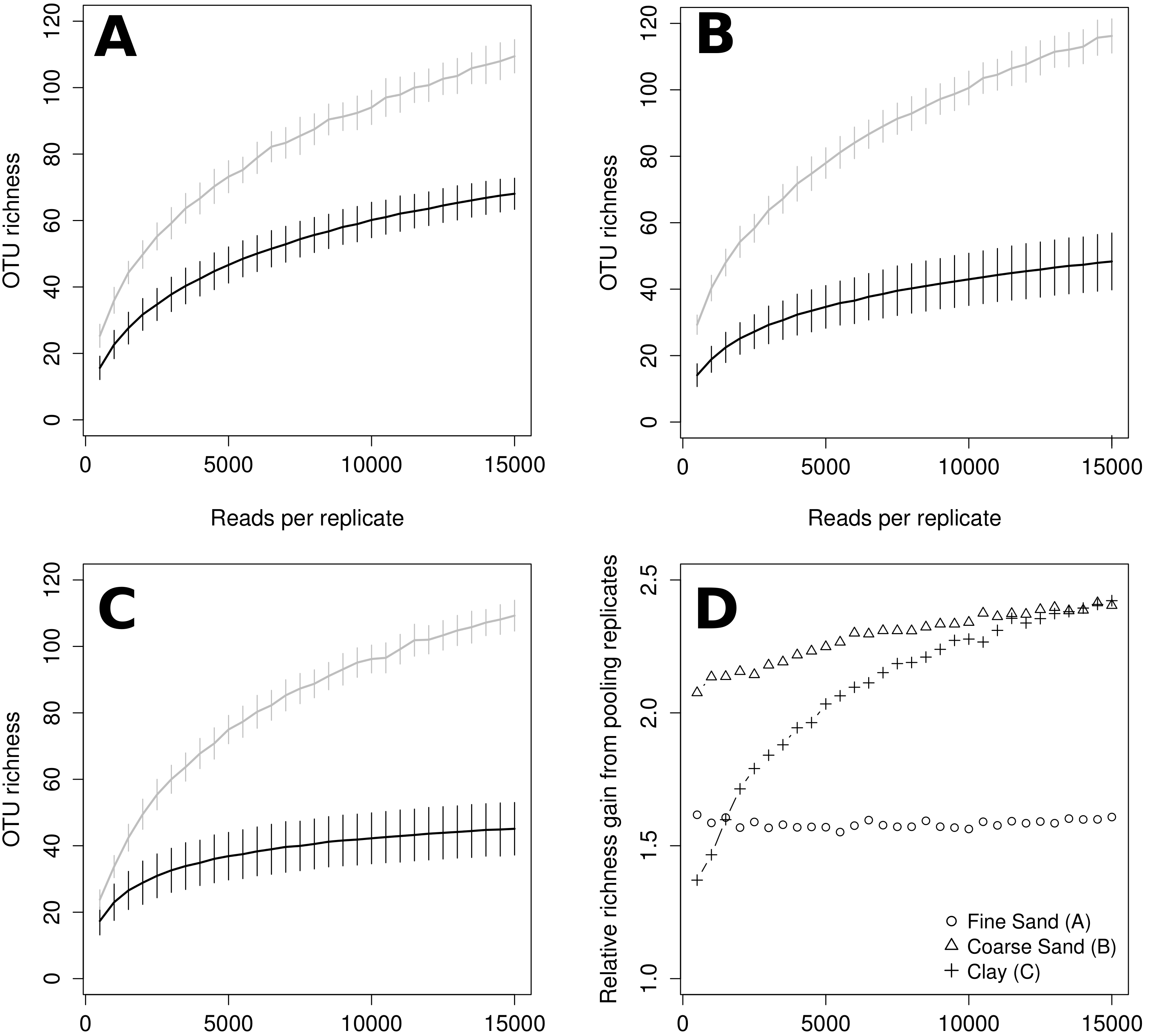

Supplement: S2 Fig — Mean and standard error of expected richness was calculated based on repeated random sub-samples at each read interval from replicates of the samples Fine Sand (A; n = 5), Coarse Sand (B; n = 7) and Clay (C, n = 5), after taxonomic filtering to include only metazoan OTUs. Panel D shows expected richness in pooled samples compared to mean expected replicate richness. (TIF) [file pone.0179443.s005.tif]

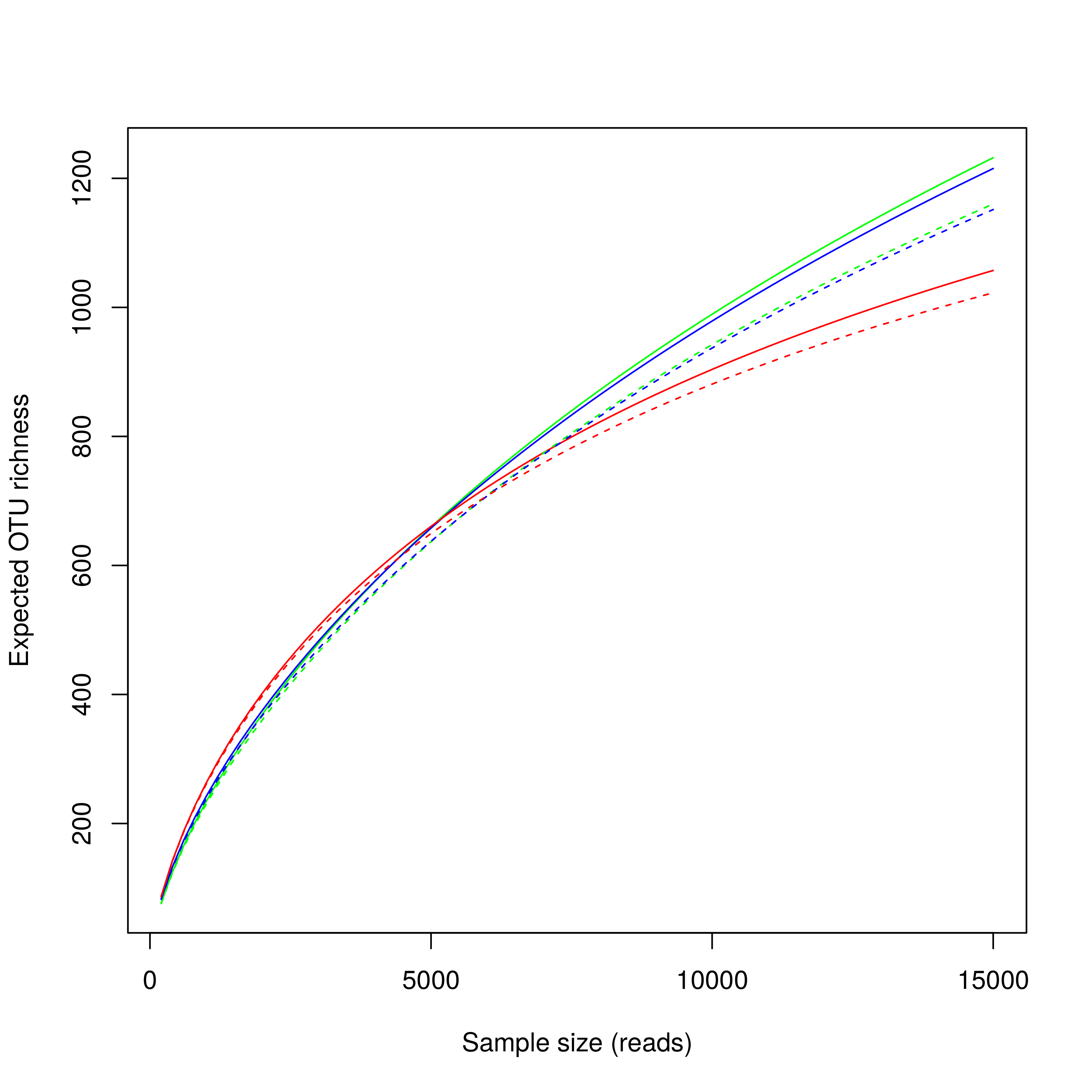

Supplement: S3 Fig — Rarefaction curves using solid lines represent the studied dataset where singletons were retained (and correspond to Figs 1 and 2), whereas dashed lines result from rarefaction curves after removing singletons. (TIF) [file pone.0179443.s006.tif]
